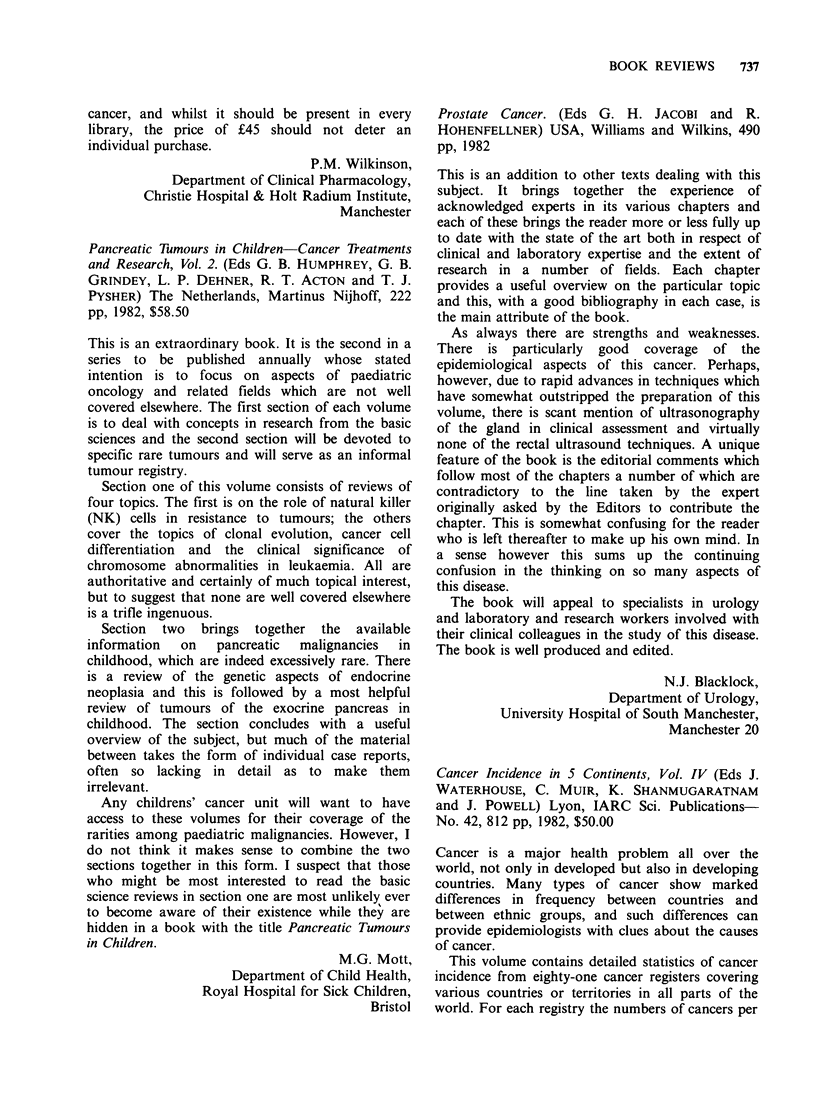# Prostate Cancer

**Published:** 1983-05

**Authors:** N.J. Blacklock


*Prostate Cancer.* (Eds G. H. JACOBI and R. HOHENFELLNER) USA, Williams and Wilkins, 490 pp, 1982

This is an addition to other texts dealing with this subject. It brings together the experience of acknowledged experts in its various chapters and each of these brings the reader more or less fully up to date with the state of the art both in respect of clinical and laboratory expertise and the extent of research in a number of fields. Each chapter provides a useful overview on the particular topic and this, with a good bibliography in each case, is the main attribute of the book.

As always there are strengths and weaknesses. There is particularly good coverage of the epidemiological aspects of this cancer. Perhaps, however, due to rapid advances in techniques which have somewhat outstripped the preparation of this volume, there is scant mention of ultrasonography of the gland in clinical assessment and virtually none of the rectal ultrasound techniques. A unique feature of the book is the editorial comments which follow most of the chapters a number of which are contradictory to the line taken by the expert originally asked by the Editors to contribute the chapter. This is somewhat confusing for the reader who is left thereafter to make up his own mind. In a sense however this sums up the continuing confusion in the thinking on so many aspects of this disease.

The book will appeal to specialists in urology and laboratory and research workers involved with their clinical colleagues in the study of this disease. The book is well produced and edited.

N.J. Blacklock,
Department of Urology,
University Hospital of South Manchester,
Manchester 20